# Fathers’ Mental Health and Children’s Aggressive Behaviour A Study Based on Data from the Norwegian Mother, Father and Child Cohort Study (MoBa)

**DOI:** 10.1007/s10578-021-01123-8

**Published:** 2021-01-28

**Authors:** Anne lise Kvalevaag, Jörg Aβmus, Eva Biringer

**Affiliations:** 1 Department of Research and Innovation, Helse Fonna Local Health Trust, P.O. Box 2170, N-5504 Haugesund, Norway; 2 Center for Clinical Research, Haukeland University Hospital, N-5021 Bergen, Norway

**Keywords:** Fathers, Prenatal psychological distress, Children, Aggressive behaviour, MoBa

## Abstract

The aim of the study was to examine the association between fathers’ prenatal psychological distress and children’s aggressive behaviour in terms of hitting others, and how children’s age, gender and temperament affect this association. The study is based on data from 20,155 fathers and mothers from the Norwegian Mother, Father and Child Cohort Study (MoBa). Fathers and mothers completed questionnaires at 17 or 18 weeks of gestation and mothers at children’s age 18 months, and 3 and 5 years. Fathers’ prenatal psychological distress was assessed by the 5-item Symptom Checklist (SCL-5), and children’s temperament by 12 items from the Emotionality Activity Sociability (EAS) Temperament Survey. Increasing prenatal psychological distress in fathers was associated with an increase of hitting from 18 months to 3 years of age in boys. Children’s temperament did not affect the association between fathers’ prenatal psychological distress and children’s aggressive behaviour.

**Supplementary Information:**

The online version contains supplementary material available at 10.1007/s10578-021-01123-8.

## Introduction

High levels of aggressive behaviour early in life predict children’s difficulties and aggressive behaviour in adolescence and later in life [1]. Knowledge about development of children’s aggressive behaviour and factors that influence this development is important for the development of preventive interventions [2]. In a review, Tremblay et al. [3] describe a biopsychosocial approach to understand and prevent the development of high level, chronic physical aggressive behaviour.

Earlier longitudinal studies indicate that physically aggressive behaviour in children, such as hitting others, is typical in the first year of life; then, for most children, this behaviour declines in the preschool years [4–6]. The development of physically aggressive behaviour is nonlinear [7]. Physically aggressive behaviour in early childhood is influenced by both genetic and environmental factors [4, 5, 8, 9]. The frequency of physically aggressive behaviour in preschool-aged children is higher in families with more than one child [2, 4]. Studies have also found sex differences in physically aggressive behaviour in children [4, 5, 9–11]. These studies have found that boys show more physically aggressive behaviour than girls in the preschool years.

A recent meta-analysis that included population-based studies demonstrated that paternal perinatal depression has a negative impact on children’s socioemotional andQuery behavioural development and that this association is more relevant in early childhood [12]. There are still gaps in the knowledge about the factors involved in the development of physically aggressive behaviour in childhood and the impact that fathers’ prenatal psychological distress may have on this development [13].

Temperament is defined as the biologically rooted individual differences in behaviour tendencies [14]; it is present early in life and is relatively stable across situations and over time [6]. The basic dimensions in temperament are described by Buss and Plomin in the emotionality-activity-sociability (EAS) model [6]. The first dimension of the EAS model, ‘emotionality’, refers to psychological instability and proneness to feelings of fear, anger and sadness. The dimension of ‘activity’ refers to tempo, vigour and endurance. ‘Sociability’ refers to the tendencies to affiliate and be responsive to others. Knowledge with regard to the impact of children’s temperament on the association between paternal psychological distress and children’s aggressive behaviour is still scarce.

In a study including 19,580 father-child dyads from the Norwegian Mother, Father and Child Cohort Study (MoBa) [15], we found that 16% of children at 5 years of age still showed physically aggressive behaviour [16]. Further, we found that high-level psychological distress in expectant fathers predicted an increased risk of hitting in their children at the age of 5 years compared with fathers who showed low-level psychological distress. However, this increased risk of hitting behaviour was partly explained by confounding variables, and when stratified for gender, the significant increased risk was found only for girls [16]. The question of whether children’s temperament moderates the association between fathers’ prenatal psychological distress and children’s hitting still needs to be answered.

The present longitudinal study explores the association between fathers’ prenatal psychological distress and children’s aggressive behaviour (hitting others) at children’s age of 18 months, 3 years and 5 years in the previously mentioned sample from the MoBa study [16]. Three research questions are addressed in the study: Is the association between fathers’ prenatal psychological distress and children’s hitting dependent on the age of the children? Does children’s temperament have an impact on the association between fathers’ prenatal psychological distress and children’s hitting? Is the association between fathers’ prenatal psychological distress and children’s hitting different for girls and boys?

## Methods

### Participants

The present study is based on data from the Norwegian Mother, Father and Child Cohort Study (MoBa) [15, 17]. The establishment of MoBa and initial data collection was based on a licence from the Norwegian Data Protection Agency and approved by the Regional Committee for Medical and Health Research Ethics. The MoBa cohort is currently regulated by the Norwegian Health Registry Act. The present study was approved by the Regional Committee of Medical and Health Research Ethics (ref. no. 2010-3204). MoBa is a prospective population-based pregnancy cohort study. The participants were recruited from all over Norway from 1999 to 2008. Pregnant women consented to participate in 40.6% of all available pregnancies. The cohort now includes 114,500 children, 95,200 mothers, and 75,200 fathers. The present study is based on version V, April 2010, of the quality-assured data files released for research on fathers’ mental health and child development. To be included in the study, each family was required to complete the following questionnaires: mothers’ questionnaire at 17 or 18 weeks of gestation, fathers’ questionnaire at 17 or 18 weeks of gestation, and mothers’ reports concerning their children at 18 months, 3 years and 5 years of age. There were 20,155 completed questionnaires for (triads of) fathers, mothers and children for these measurement points.

## Measures

### Predictor Variables

Fathers’ mental health in pregnancy weeks 17 or 18 was assessed by the 5-item Symptom Checklist (SCL-5) [18, 19]. The SCL-5 is a screening measure of psychological distress that is used as an indicator of global mental distress. The SCL-5 consists of five items: (1) ‘Feeling fearful’, (2) ‘Nervousness or shakiness inside’, (3) ‘Feeling hopeless about the future’, (4) ‘Feeling blue’ and (5) ‘Worrying too much about things’. Each of the five items is scored on a scale from 1 to 4, depending on how bothered the respondent has been in that area during the 14 days prior to the time of self-report: 1 = ’Not bothered’, 2 = ’A little bothered’, 3 = ’Quite bothered’, and 4 = ’Very bothered’. The checklist mainly screens for symptoms of anxiety and depression [20]. Descriptive characteristics of the mean scale are shown in Table 1. Intra-scale consistency as estimated by Cronbach’s alpha was 0.79. The customary cut-off of 2.00 [19] for case-level psychological distress was used.

**Table 1:** Descriptive characteristics of fathers and mothers reported at weeks 17 to 18 of pregnancy (N = 20,155)

Characteristics	Fathers N (%)	Mothers N (%)
Age		
≤ 19	48 (0.2%)	145 (0.7%)
20–24	889 (4.4%)	2110 (11%)
25–29	5142 (26%)	7429 (37%)
30–34	8151 (40%)	7684 (38%)
35–39	4267 (21%)	2513 (13%)
40–44	1220 (6%)	274 (1.4%)
45–49	335 (1.7%)	0 (0%)
≥ 50	103 (0.5%)	0 (0%)
Missing	0 (0%)	0 (0%)
Marital status		
Married	10,650 (53%)	10,661 (53%)
Co-habiting	9079 (45%)	9080 (45%)
Single	150 (0.7%)	152 (0.8%)
Divorced/separated	49 (0.2%)	31 (0.2%)
Other	147 (0.7%)	179 (0.9%)
Missing	80 (0.4%)	52 (0.3%)
Education		
Secondary education	804 (4%)	361 (1.8%)
1–2 years further education	1248 (6%)	877 (5%)
3 years further education	7713 (38%)	5310 (26%)
Higher education (university/college) ≤ 4 years	5422 (27%)	8575 (43%)
Higher education (university/college) ≥ 4 years	4175 (21%)	4081 (20%)
Missing	793 (3.9%)	951 (4.7%)
Daily cigarette smoking		
No	10, 681 (53%)	10,437 (52%)
Yes	3797(19%)	8860 (44%)
Smoked earlier, current status unknown	2948 (15%)	NA^a^
Missing	2729 (14%)	858 (4%)
Use of alcohol		
Never/less than once a month	5463 (27%)	17,140 (85%)
Once a week or less	11,887 (59%)	617 (3%)
≥ 2 times/ week	2176 (11%)	16 (0.1%)
Missing	629 (3%)	2382 (12%)
Physical activity		
0–2 times a week	6807 (34%)	6702 (33%)
≥ 3 times a week	13,181 (65%)	10,586 (53%)
Missing	167 (0.8%)	2867 (14%)
Somatic condition	3877 (19%)	3786 (19%)
Employment status^b^		
Student	953 (5%)	1637 (8%)
Out of work	465 (2.3%)	528 (3%)
Domestic work	105 (0.5%)	1182 (6%)
Employed work or self-employed	19,524 (97%)	17,475 (87%)
Disability pension	263 (1.3%)	285 (1.4%)
Military service	49 (0.2%)	2 (0.0%)
Other	607 (3%)	707 (4%)
Missing	56 (0.3%)	81 (0.4%)
Pregnancy planned		
No		3167 (16%)
Yes		16,753 (83%)
Missing		235 (1.2%)
SCL-5^c^ dichotomised		
< 2	19,451 (97%)	19,040 (95%)
≥ 2	551 (3%)	1108 (6%)
Missing	153 (0.8%)	7 (0.0%)

	Mean (SD^d^), range	Mean (SD^d^), range
SCL-5	1.12 (0.28), 1–4	1.27 (0.33), 1–4
	(153 missing)	(7 missing)
MSS-10^e^	53.4 (5.56), 10–60	52.7 (6.68), 10–60
	(152 missing)	(3021 missing)

^a^Information not available

^b^Numbers do not add up to 100% as some participants were included in more than one category

^c^5-items symptom checklist

^d^Standard deviation

^e^10-items marital satisfaction scale

### Outcome Variables

At children’s age 18 months and 3 and 5 years, mothers were asked to respond to several of the items from the Child Behavioural Checklist Revised (CBCL-R) [20–22], including the child hitting others. The item was rated on a 3-point scale: 0 = ’Not true’, 1 = ’Somewhat/sometimes true’ and 2 = ’Very true/often true’. The responses were dichotomised with children’s hitting others reported as ‘Sometimes true’ and ‘Often true’ in one category (= 1) and ‘Not true’ (= 0) in the other.

### Moderating Variables

**Fig. 1:**
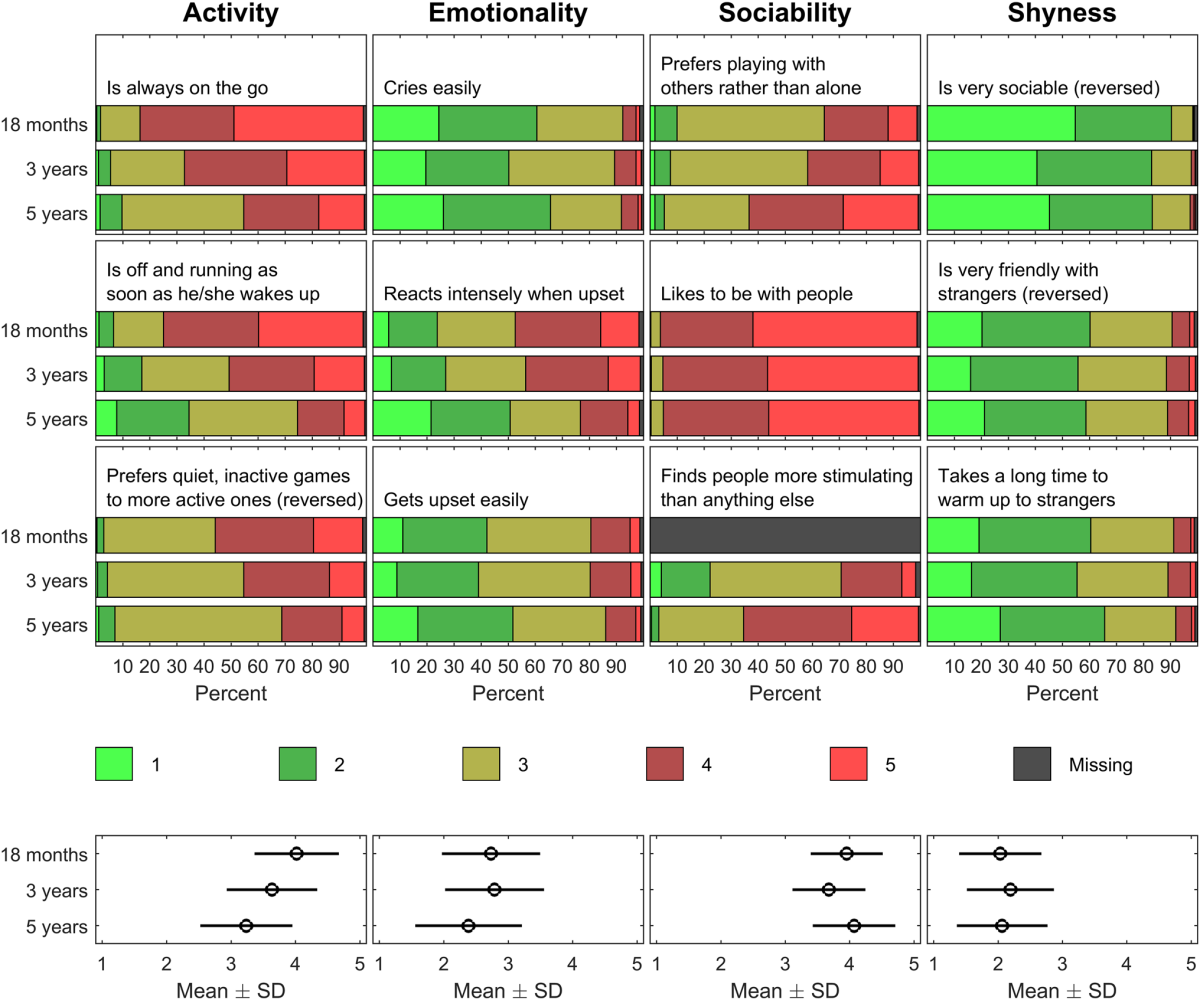
Children’s temperament: Percentages of Emotionality Activity Sociability (EAS) Temperament Survey item responses and averaged subscales with standard deviations (SD) at ages 18 months, three years and 5 years

Dagitty is a graphical tool for analysing causal diagrams, and it is also known as directed acyclic graphs (DAGs). To identify the variables that the statistical models should be adjusted for, the following relevant constructs were entered into a dagitty diagram: fathers’ age, education, marital status, somatic condition, cigarette smoking, prenatal psychological distress, marital satisfaction and whether they lived with the family; mothers’ age, prenatal psychological distress, marital satisfaction, daily care of the children (kindergarten), number of children in household and whether the pregnancies were planned; children’s temperament, language development and whether they hit others. According to the resulting dagitty diagram, the models should be adjusted for children’s temperament, children’s language development, number of children in household, daily care, whether fathers were living with family, fathers’ age, mothers’ age, and mothers’ prenatal psychological distress. Descriptive statistics of these variables are shown in Fig. 1 and Tables 1 and 2.

**Table 2:** Characteristics of the children and their surroundings at age 18 months, 3 and 5 years (N = 20,155)

Characteristic	18 months N (%)	3 years N (%)	5 years N (%)
Children hitting others			
Often hitting	354 (1.8)	323 (1.6)	153 (0.8)
Sometimes hitting	6875 (34)	7,111 (35)	3029 (15)
Not hitting	12, 812 (64)	12, 501 (62)	16,833 (84)
Missing	474 (0.6)	220 (1.1)	140 (0.7)
Boys hitting others			
N = 10,279 (51%)			
Often hitting	226 (2.2)	217 (2.1)	77 (0.7)
Sometimes hitting	3829 (37)	4303 (42)	1575 (15)
Not hitting	6167 (60)	5650 (55)	8556 (83)
Missing	57 (0.6)	109 (1.1)	71 (0.7)
Girls hitting others			
N = 9876 (49%)			
Often hitting	128 (1.3)	106 (1.1)	76 (0.8)
Sometimes hitting	3046 (31)	2808 (29)	1454 (15)
Not hitting	6645 (67)	6851 (69)	8277 (84)
Missing	57 (0.6)	111 (1.1)	69 (0.7)
Somatic conditions			
Yes	3083 (15)	4312 (21)	3852 (19)
Language problems^a^			
Yes	212 (1.1)	537 (2.7)	1281 (6)
No	19,573 (97)	19,118 (95)	18,721 (93)
Missing	370 (1.8)	500 (2.5)	153 (0.8)
Number of children in household			
1	6,756 (34)	5,097 (25)	3,706 (18)
2	8,198 (41)	9,070 (45)	9,877 (49)
3	2,986 (15)	4,032 (20)	4,862 (25)
4	615 (3.1)	747 (3.7)	922 (4.6)
≥ 5	143 (0.7)	176 (0.9)	223 (1.1)
Missing/non-valid	1457 (7.2)	1033 (5.1)	565 (2.8)
Mother and father living together			
Yes	12,514 (62)	18,632 (92)	18,278 (91)
No	499 (2.5)	824 (4)	1,579 (8)
Never	NA^b^	NA^b^	226 (1.1)
Missing	7142 (35)	699 (3.5)	72 (0.4)
Daily care			
Kinder-garden	9208 (46)	7451 (37)	14,528 (72)

^a^Delayed or defiant language development as reported by mother at the time of measurement on two items of the Ages and Stages Questionnaire (ASQ)

^b^Information not available

### Operationalisation of Children’s Temperament

The MoBa included 12 of the original 20 items constituting the Emotionality Activity Sociability (EAS) Temperament Survey [21] at children’s age 18 months, 3 years and 5 years. The 12 items represented the original ‘Emotionality’ (three items), ‘Activity’ (three items), ‘Shyness’ (three items) and ‘Sociability’ (three items) subscales of the EAS. In the Norwegian version of the instrument, the item ‘Makes friends easily’ was rephrased into ‘Enjoys being with other children’ [21]. The original MoBa items were scaled from 1 to 5, with 1 representing ‘Very characteristic or typical of your child’, and 5 meaning ‘Not characteristic or typical of your child’. Nine of the 12 items were reverse-scaled so that for all items, 1 corresponded to low temperament and 5 to high temperament. Figure 1 shows the EAS item frequencies at 18 months and 3 and 5 years of age.

The original EAS subscales each included five items that added up to produce scales ranging from 5 to 25. In the present study, four averaged subscales representing children’s activity, emotionality, sociability and skewness were computed at each point of measurement by adding up the three items from each of these EAS dimensions available in MoBa and then dividing the sum of these items by the number of available items within each subscale. The means and standard deviations (SDs) of these subscales representing children’s temperament are shown in the lower part of Fig. 1. Inter-item reliability as assessed by Cronbach’s alpha ranged from 0.64 to 0.66 for the activity, 0.63 to 0.75 for the emotionality, 0.32 to 0.71 for the sociability and 0.65 to 0.69 for the shyness subscales across children’s age of 18 months, 3 years and 5 years.

### Marital Satisfaction Scale (MSS)

The MoBa questionnaire included the 10-item MSS [22]. Examples of items are ‘My partner and I have problems in our relationship’, ‘I am very happy in my relationship’, ‘I am satisfied with my relationship with my partner’; and ‘We agree about how children should be raised’. Each of the items was scored on a 6-point scale: 6 = ‘Totally agree’, 5 = ‘Agree’, 4 = ‘Slightly agree’, 3 = ‘Slightly disagree’, 2 = ‘Disagree’ and 1 = ‘Totally disagree’. Negatively worded items were reverse-scaled. A summary scale was created by adding up the item scores (Table 1). Higher scores on the summary scale indicated a more positive relationship with the partner. Internal scale consistency, as assessed by Cronbach’s alpha in pregnancy, was 0.88 for fathers’ and 0.91 for mothers’ MSS.

### Mothers’ Psychological Distress (SCL-5)

Descriptive statistics of mothers’ SCL-5 mean scale in pregnancy are shown in Table 1. Intra-scale consistency in pregnancy as estimated by Cronbach’s alpha 0.57 for mothers’ SCL-5 total scale. Mothers’ mean SCL-5 scores were *M* = 1.3, SD = 0.39, range 1–4, *M* = 1.3, SD = 0.41, range = 1–4, and *M* = 1.2, SD = 0.37, range 1–4 at children’s age 18 months, 3 years and 5 years, respectively.

### Children’s Language Skills

We included two items from the Ages and Stages Questionnaire (ASQ) [23]. The ASQ is a screening instrument for child development from four to 60 months of age based on parents’ reports [24]. It contains 30 items, with each item scored on a 3-point scale: 1 = ‘Yes’, 2 = ‘A few times’ and 3 = ‘Not yet’. Two items are used in the present analysis (1) ‘Without giving him/her help by pointing or using gestures, ask your child to put the shoe on the table and put the book under the chair. Does your child carry out both of these directions correctly?’ (impressive language skills) and (2) ‘Can your child tell you at least two things about an object he/she is familiar with? If you say, for example, tell me about your ball, will your child answer by saying something like, It is round, I can throw it, it is big? ‘ (expressive language skills).

### Data Analysis

Repeated measures data have a hierarchical structure which can be analysed using multilevel models. The effect of children’s temperament on the association between fathers’ prenatal psychological distress and children’s hitting was therefore investigated using linear mixed effects models (LMEs). Analyses were done in three steps. First, we assessed the association without children’s temperament by using the unadjusted LME model for children’s hitting depending on fathers’ SCL-5 in pregnancy and children’s age, as well as their interaction, with a random individual intercept and simple contrasts over time. Second, we adjusted the first model for each of the four EAS temperament scales to study the effect of these adjustments on the model parameters. Finally, we adjusted each of the four EAS-adjusted models successively for the number of children in the household, daily care (kindergarten), children’s language skills and further characteristics. Further characteristics were entered as a block consisting of fathers’ and mothers’ age, mothers’ SCL-5 and whether fathers were living with the family. These adjustment variables were selected using DAGs. The models were tested in three steps and compared using Analysis of Variance (ANOVA). This required the same number of observations in each step. This is why we used the cases available in all steps to compare the models and all available observations to interpret the models. All analyses were done both for the total sample of all children and stratified for children’s gender.

The computation was done in the nlme package of R 3.6 (R Core team [25]) and IBM SPSS 23 (IBM Corp., Armonk, NY), and graphics were created using Matlab 9.0 (Mathworks, Natick, MA). Tests were two-tailed with a significance level of 0.05.

## Results

### Association of fathers’ prenatal psychological distress with children’s age and children’s hitting

**Fig. 2:**
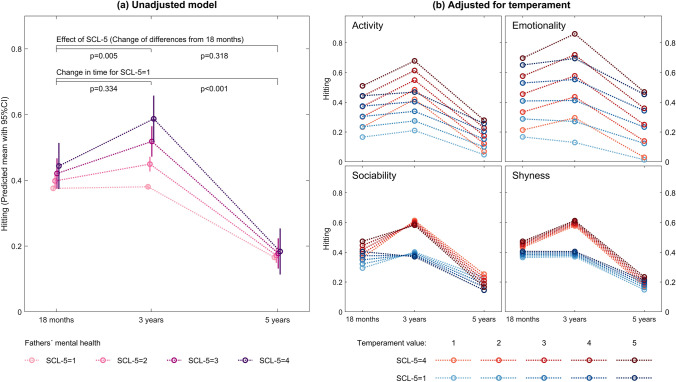
Hitting depending on fathers’ prenatal Symptom Checklist (SCL-5), children’s age and their interaction. Predicted mean with 95% confidence interval (CI). Crude models and models adjusted for children’s temperament in terms of emotionality, activity, sociability (EAS) temperament survey subscales

The associations of children’s age and hitting for different levels of fathers’ prenatal psychological distress as measured by SCL-5 in pregnancy are shown in Fig. 2. Without adjusting for children’s temperament, we observed that for fathers without prenatal psychological distress (i.e., SCL-5 = 1), there was no change in children’s hitting from children’s age 18 months to 3 years, while children’s hitting decreased significantly from 18 months to 5 years (p < 0.001) (Table 3). With increasing SCL-5 scores in fathers, children’s hitting increased significantly from children’s age 18 months to 3 years (B = 0.046, 95% confidence interval (CI) [0.014, 0.079], p = 0.005) and decreased to the same level as for fathers without prenatal psychological distress (i.e., SCL-5 = 1) again at 5 years (Fig. 2a).

**Table 3:** Linear mixed effects model with fathers' SCL-5 in pregnancy and children's age, and their interaction, as predictor variables and children's hitting as outcome variable (N = 18,955)

Predictors	All children		Girls		Boys	
	B (95% CI)	p-value	B (95% CI)	p-value	B (95% CI)	p value
0-model (N = 19,094)						
Children's age	–	–	–	–	–	–
18 m ≥ 3 year	0.011 [0.002, 0.02]	0.020	− 0.027 [− 0.039, − 0.015]	< 0.001	0.047 [0.034, 0.06]	< 0.001
18 m ≥ 5 year	− 0.212 [− 0.221, − 0.203]	< 0.001	− 0.173 [− 0.185, − 0.16]	< 0.001	− 0.250 [− 0.262, − 0.237]	< 0.001
Unadjusted model [N = 18,955]						
Children's age	–	–	–	–	–	–
18 m ≥ 3 year	0.005 [− 0.005, 0.015]	0.334	− 0.033 [− 0.046, − 0.019]	< 0.001	0.041 [0.027, 0.055]	< 0.001
18 m ≥ 5 year	− 0.210 [− 0.22, − 0.2]	< 0.001	− 0.171 [− 0.185, − 0.158]	< 0.001	− 0.248 [− 0.262, − 0.234]	< 0.001
SCL-5 father	0.022 [− 0.002, 0.047]	0.070	0.026 [− 0.008, 0.06]	0.136	0.017 [− 0.017, 0.051]	0.327
Interaction fathers’ SCL-5*children's age	–	–	–	–	–	–
18 m ≥ 3 year	0.046 [0.014, 0.079]	0.005	0.043 [− 0.004, 0.089]	0.070	0.047 [0.001, 0.093]	0.045
18 m ≥ 5 year	− 0.017 [− 0.049, 0.016]	0.318	− 0.018 [− 0.063, 0.028]	0.453	− 0.013 [− 0.059, 0.033]	0.568
Model adjusted for emotionality						
Children’s age	–	–	–	–	–	–
18 m ≥ 3 year	− 0.002 [− 0.011, 0.008]	0.759	− 0.045 [− 0.058, − 0.031]	< 0.001	0.041 [0.027, 0.055]	< 0.001
18 m ≥ 5 year	− 0.168 [− 0.178, − 0.158]	< 0.001	− 0.133 [− 0.146, − 0.119]	< 0.001	− 0.201 [− 0.216, − 0.187]	< 0.001
SCL5 father	0.015 [− 0.009, 0.039]	0.223	0.014 [− 0.02, 0.048]	0.431	0.014 [− 0.02, 0.048]	0.428
Interaction fathers’ SCL-5*children's age	–	–	–	–	–	–
18 m ≥ 3 year	0.042 [0.009, 0.075]	0.012	0.040 [− 0.007, 0.087]	0.098	0.040 [− 0.006, 0.086]	0.089
18 m ≥ 5 year	− 0.010 [− 0.042, 0.023]	0.555	− 0.005 [− 0.052, 0.042]	0.838	− 0.011 [− 0.057, 0.035]	0.648
Model adjusted for activity						
Children's age	–	–	–	–	–	–
18 m ≥ 3 year	0.029 [0.019, 0.039]	< 0.001	− 0.011 [− 0.025, 0.003]	0.122	0.064 [0.05, 0.079]	< 0.001
18 m ≥ 5 year	− 0.162 [− 0.173, − 0.151]	< 0.001	− 0.132 [− 0.146, − 0.117]	< 0.001	− 0.198 [− 0.213, − 0.182]	< 0.001
SCL5 father	0.022 [− 0.003, 0.046]	0.081	0.026 [− 0.008, 0.06]	0.136	0.015 [− 0.019, 0.05]	0.388
Interaction fathers’ SCL-5*children’s age	–	–	–	–	–	–
18 m ≥ 3 year	0.048 [0.015, 0.081]	0.004	0.043 [− 0.003, 0.09]	0.067	0.049 [0.003, 0.096]	0.037
18 m ≥ 5 year	− 0.015 [− 0.047, 0.018]	0.385	− 0.016 [− 0.062, 0.03]	0.503	− 0.011 [− 0.057, 0.035]	0.647
Model adjusted for shyness						
Children’s age	–	–	–	–	–	–
18 m ≥ 3 year	0.003 [− 0.007, 0.013]	0.557	− 0.033 [− 0.046, − 0.019]	< 0.001	0.037 [0.022, 0.051]	< 0.001
18 m ≥ 5 year	− 0.211 [− 0.221, − 0.201]	< 0.001	− 0.171 [− 0.185, − 0.157]	< 0.001	− 0.251 [− 0.265, − 0.236]	< 0.001
SCL5 father	0.022 [− 0.002, 0.047]	0.077	0.027 [− 0.008, 0.061]	0.126	0.015 [− 0.019, 0.05]	0.381
Interaction fathers’ SCL-5*children's age	–	–	–	–	–	–
18 m ≥ 3 year	0.047 [0.014, 0.08]	0.005	0.040 [− 0.007, 0.086]	0.094	0.050 [0.003, 0.096]	0.035
18 m ≥ 5 year	− 0.015 [− 0.048, 0.018]	0.379	− 0.017 [− 0.063, 0.029]	0.473	− 0.010 [− 0.057, 0.036]	0.666
Model adjusted for sociability						
Children’s age	–	–	–	–	–	–
18 m ≥ 3 year	0.004 [− 0.006, 0.014]	0.401	− 0.031 [− 0.045, − 0.017]	< 0.001	0.039 [0.025, 0.054]	< 0.001
18 m ≥ 5 year	− 0.210 [− 0.22, − 0.2]	< 0.001	− 0.172 [− 0.186, − 0.158]	< 0.001	− 0.247 [− 0.261, − 0.233]	< 0.001
SCL5 father	0.023 [− 0.002, 0.047]	0.070	0.027 [− 0.007, 0.061]	0.123	0.016 [− 0.019, 0.051]	0.363
Interaction fathers’ SCL-5*children's age	–	–	–	–	–	–
18 m ≥ 3 year	0.048 [0.015, 0.081]	0.005	0.039 [− 0.008, 0.085]	0.102	0.054 [0.007, 0.1]	0.024
18 m ≥ 5 year	− 0.016 [− 0.049, 0.017]	0.332	− 0.018 [− 0.064, 0.029]	0.456	− 0.012 [− 0.059, 0.034]	0.600

### Impact of children’s temperament on the association between fathers’ prenatal psychological distress and children’s hitting

As shown in Table 3, Fig. 2 and Online Resource 1, the association of fathers’ prenatal psychological distress and children’s age with children’s hitting was unaffected by children’s temperament, with one exception. We observed a significant increase in children’s hitting also for fathers without psychological distress (B = 0.029, 95% CI [0.019, 0.039], p < 0.001) when adjusting for the EAS ‘Activity’ subscale. However, the associations between the interaction terms of fathers’ SCL-5 with children’s age were not affected by adjusting for the ‘Emotionality’, ‘Shyness’ and ‘Sociability’ subscales. The coefficients for the association of fathers’ prenatal psychological distress and change in children’s hitting from children’s age 18 months to 3 years (0.042–0.048) varied within the 95% CI of the unadjusted model, while the associations of fathers’ prenatal psychological distress and change in children’s hitting at children’s age 18 months and changes from 18 months to 5 years remained nonsignificant after adjusting for children’s temperament. Adjusting for other family- and parent-related variables did not lead to an essential change in the coefficients for the associations of fathers’ prenatal psychological distress and children’s age with children’s hitting.

### Impact of children’s gender on the association between fathers’ prenatal psychological distress and children’s hitting

The LME model, as stratified by children’s gender, is shown in Table 3. For boys, we observed an increase in children’s hitting from children’s age 18 months to 3 years (B = 0.041, 95% CI [0.027, 0.055], p < 0.001). This increase was independent of fathers’ prenatal SCL-5. Otherwise, the same pattern as described for all children was found in boys. For girls, the hitting decreased significantly, both from 18 months to 3 years (B =  − 0.033, 95% CI [− 0.046, − 0.019], p < 0.001) and 5 years (B =  − 0.171, 95% CI [− 0.185, − 0.158], p < 0.001).

For increasing prenatal psychological distress in fathers, we observed an estimated increase of hitting from children’s age 18 months to 3 years in boys (B = 0.047, 95% CI [0.001, 0.093], p = 0.045). With increasing prenatal psychological distress in fathers there was also a descriptive, but nonsignificant, increase in hitting for girls from 18 months to 3 years of age (B = 0.043, 95% CI [− 0.004, 0.089], p = 0.070).

## Discussion

We found that higher levels of psychological distress in fathers in pregnancy were associated with a larger increase in hitting in their children from 18 months to 3 years of age. The association was statistically significant for boys, and a trend for girls. Children with fathers with low levels of prenatal psychological distress did not show significant changes in hitting from 18 months to 3 years of age. There was no difference between the levels of fathers’ prenatal psychological distress with regard to children’s hitting when the children had reached 5 years. Notably, children’s temperament did not affect the association between fathers’ prenatal psychological distress and increase in children’s hitting.

This finding that the increase in children’s hitting from 18 months to 3 years of age was significantly larger for higher levels of fathers’ prenatal psychological distress may reflect an effect of fathers’ prenatal psychological distress on children’s hitting via fathers’ postnatal mental health [24]. A genetically transmitted risk from fathers to the children may also explain some of the association between fathers’ prenatal psychological distress and children’s increase in hitting [26]. Further, psychological distress in expecting fathers may have an impact on the mental health of their pregnant partners, and therefore indirectly have negative effect on children’s outcomes through an impact on the mothers [27]. This is in accordance with the review by Tremblay et al. [3] stating that there are interrelated biopsychosocial ‘channels’ involved in the development of physical aggressive behaviour.

The findings of higher increase of physically aggressive behaviour in children with more paternal distress, confirms findings in an earlier study [7]. The overall decrease in hitting in children from 3 years of age to 5 years of age in the present study is also in line with other studies [4, 5, 7, 13, 16]. Our findings also confirm earlier studies demonstrating that physical aggressive behaviour takes a nonlinear form [7]. In the present study, children’s hitting reached the highest point at 3 years of age and then declined until 5 years of age.

Our finding that children’s temperament did not moderate the association between fathers’ psychological distress and children’s hitting is not in line with the results by Nærde et al. [7], who found support for the overall significance of children’s temperament for the early use of physical aggressive behaviour in infancy. However, their study was not directly comparable to ours in terms of the time points of measurement and operationalisation of constructs investigated, and these differences between studies may explain the divergent findings.

In the present study, we observed an increasing frequency of hitting from 18 months to 3 years in boys. The increase in hitting in boys in the present study is in line with Nærde et al. [7], who found that boys showed somewhat higher levels of physical aggressive behaviour than girls; they stated that earlier findings are unclear as to how early gender differences appear and whether the magnitude of the differences increases over time [28, 29]. In our earlier study using the same sample as in the present study, we observed an increase in hitting from 18 months to 3 years of age for both boys and girls with increasing psychological distress in fathers, but this increase was only significant for girls. Children’s hitting in our previous study was, however, operationalised as; stopped hitting before 5 years of age and hitting at 5 years of age versus never hitting [16]. These previous findings also imply that the trajectories of aggressive behaviour differ between genders and that fathers’ psychological distress impacts boys and girls differently. However, the overall picture of the impact of gender on physical aggressive behaviour is still unclear [4, 11], and further studies are needed.

### Strengths and Limitations

The present study was population-based with a large sample size and, as such, has the statistical power to detect even weak associations between fathers’ prenatal psychological distress and children’s, age, temperament and hitting. By testing these aspects of the association between fathers’ mental health and children’s aggressive behaviour in a population sample, the serious selection biases that are commonly found in clinical studies have probably been avoided. However, we cannot exclude inclusion biases as limitations. The study participants may differ from nonparticipants in ways that are not random with respect to the exposure and outcome [30, 31]. However, Nilsen et al. [30] previously concluded that only the prevalence estimates of exposure and outcome, not the exposure–outcome associations, were biased because of self-selection in MoBa [30]. Further, the prospective design of the study is a strength because it makes a child-to-parent effects less likely as the cause of the associations found. Rather, the associations found represent evidence of parent-to-child directionality. Some limitations regarding the information gathered and operationalisation of the measures need to be mentioned. First, all data were self-reported by fathers or mothers and, thus, are subject to reporting biases, possibly limiting the reliability of the findings. Second, the reliability of the findings could have been higher if complete valid clinical instruments were used. Unfortunately, only 12 of the original 20 items of the EAS were available in the MoBa and the construct reliability of the measure may be reduced. We chose to measure hitting as outcome variable because it is a concrete type of physically aggressive behaviour, which makes it relatively easy for the parents to discern and quantify. However, the outcome variable ‘hitting’ had only three response categories and, therefore, a limited variance. The restriction of variance on the outcome variable could also lead to the weakening of the associations studied. Finally, the lack of postnatal information about fathers’ psychological distress in the MoBa study limits the ability to draw causal inferences about the effects of fathers’ postnatal psychological distress on their children’s hitting.

## Summary

Our study adds to the understanding of the impact of fathers’ prenatal psychological distress on children’s physical aggressive behaviour. Boys with fathers with high levels of prenatal psychological distress showed a greater increase in hitting than boys whose fathers had low levels of prenatal psychological distress. This development happened independently of children’s temperament. The development of physical aggressive behaviour may be influenced by both genetic and environmental factors through interrelated channels from conception onwards. Further studies on the predictors and development of physical aggressive behaviour in children are needed. Future studies may inform preventive interventions in pregnancy and early life.

## Supplementary Information

Below is the link to the electronic supplementary material.

Supplementary file1 (DOCX 20 KB)
