# Supplementary material for: Fathers’ Mental Health and Children’s Aggressive Behaviour A Study Based on Data from the Norwegian Mother, Father and Child Cohort Study (MoBa)
Source: Child Psychiatry Hum Dev. 2021 Jan 28;53(2):278–88. doi: 10.1007/s10578-021-01123-8 (PMC8924092; doi:10.1007/s10578-021-01123-8)
Supplement: Supplementary file 1 — Supplementary file1 (DOCX 20 KB) [file 10578_2021_1123_MOESM1_ESM.docx]

Online Resource 1 Linear mixed effects model with fathers' SCL-5 in pregnancy and children's age, and their interaction, as predictor variables and children's hitting as outcome variable. Unadjusted and adjusted models.

| **Children's Emotionality** | | | | **Children's Activity** | | | | **Children's Shyness** | | | | **Children's Sociability** | | | |
| --- | --- | --- | --- | --- | --- | --- | --- | --- | --- | --- | --- | --- | --- | --- | --- |
| **Estimates** | | **ANOVA** | | **Estimates** | | **ANOVA** | | **Estimates** | | **ANOVA** | | **Estimates** | | **ANOVA** | |
| **B**  **(95%CI)** | **p-value** | **AIC** | **p-value** | **B**  **(95%CI)** | **p-value** | **AIC** | **p-value** | **B**  **(95%CI)** | **p-value** | **AIC** | **p-value** | **B**  **(95%CI)** | **p-value** | **AIC** | **p-value** |
| - | - | 77227 | - | - | - | 79647 | - | - | - | 79996 | - | - | - | 79941 | - |
| -0.002  (-0.011, 0.008) | 0.759 | - | - | 0.029  (0.019, 0.039) | <0.001 | - | - | 0.003  (-0.007, 0.013) | 0.557 | - | - | 0.004  (-0.006, 0.014) | 0.401 | - | - |
| -0.168  (-0.178, -0.158) | <0.001 | - | - | -0.162  (-0.173, -0.151) | <0.001 | - | - | -0.211  (-0.221, -0.201) | <0.001 | - | - | -0.21  (-0.22, -0.2) | <0.001 | - | - |
| 0.015  (-0.009, 0.039) | 0.223 | 77218 | <0.001 | 0.022  (-0.003, 0.046) | 0.081 | 79632 | <0.001 | 0.022  (-0.002, 0.047) | 0.077 | 79980 | <0.001 | 0.023  (-0.002, 0.047) | 0.07 | 79925 | <0.001 |
| - | - | 77212 | 0.004 | - | - | 79621 | <0.001 | - | - | 79970 | <0.001 | - | - | 79914 | <0.001 |
| 0.042  (0.009, 0.075) | 0.012 | - | - | 0.048  (0.015, 0.081) | 0.004 | - | - | 0.047  (0.014, 0.08) | 0.005 | - | - | 0.048  (0.015, 0.081) | 0.005 | - | - |
| -0.01  (-0.042, 0.023) | 0.555 | - | - | -0.015  (-0.047, 0.018) | 0.385 | - | - | -0.015  (-0.048, 0.018) | 0.379 | - | - | -0.016  (-0.049, 0.017) | 0.332 | - | - |
| **N=18461, AIC=73063, p=0.005** | | | | **N=18464, AIC=75363, p=0.021** | | | | **N=18464, AIC=75676, p=0.036** | | | | **N=18464, AIC=75635, p=0.032** | | | |
| **Estimates** | | **ANOVA** | | **Estimates** | | **ANOVA** | | **Estimates** | | **ANOVA** | | **Estimates** | | **ANOVA** | |
| **B**  **(95%CI)** | **p-value** | **AIC** | **p-value** | **B**  **(95%CI)** | **p-value** | **AIC** | **p-value** | **B**  **(95%CI)** | **p-value** | **AIC** | **p-value** | **B**  **(95%CI)** | **p-value** | **AIC** | **p-value** |
| - | - | 73087 | - | - | - | 75393 | - | - | - | 75705 | - | - | - | 75665 | - |
| -0.003  (-0.013, 0.007) | 0.522 | - | - | 0.027  (0.017, 0.038) | <0.001 | - | - | 0.001  (-0.009, 0.012) | 0.808 | - | - | 0.003  (-0.008, 0.013) | 0.606 | - | - |
| -0.168  (-0.179, -0.158) | <0.001 | - | - | -0.162  (-0.173, -0.15) | <0.001 | - | - | -0.211  (-0.221, -0.2) | <0.001 | - | - | -0.209  (-0.22, -0.199) | <0.001 | - | - |
| 0.015  (-0.01, 0.04) | 0.237 | 73077 | <0.001 | 0.022  (-0.003, 0.048) | 0.085 | 75378 | <0.001 | 0.023  (-0.003, 0.048) | 0.081 | 75690 | <0.001 | 0.023  (-0.002, 0.049) | 0.074 | 75649 | <0.001 |
| - | - | 73069 | 0.002 | - | - | 75367 | <0.001 | - | - | 75679 | <0.001 | - | - | 75637 | <0.001 |
| 0.045  (0.011, 0.079) | 0.009 | - | - | 0.049  (0.015, 0.083) | 0.005 | - | - | 0.048  (0.014, 0.082) | 0.005 | - | - | 0.049  (0.015, 0.083) | 0.005 | - | - |
| -0.01  (-0.044, 0.023) | 0.55 | - | - | -0.015  (-0.049, 0.018) | 0.372 | - | - | -0.016  (-0.049, 0.018) | 0.365 | - | - | -0.017  (-0.051, 0.016) | 0.314 | - | - |
| **N=18461, AIC=73062, p=0.051** | | | | **N=18464, AIC=75362, p=0.082** | | | | **N=18464, AIC=75673, p=0.021** | | | | **N=18464, AIC=75632, p=0.032** | | | |
| **Estimates** | | **ANOVA** | | **Estimates** | | **ANOVA** | | **Estimates** | | **ANOVA** | | **Estimates** | | **ANOVA** | |
| **B**  **(95%CI)** | **p-value** | **AIC** | **p-value** | **B**  **(95%CI)** | **p-value** | **AIC** | **p-value** | **B**  **(95%CI)** | **p-value** | **AIC** | **p-value** | **B**  **(95%CI)** | **p-value** | **AIC** | **p-value** |
| - | - | 73087 | - | - | - | 75393 | - | - | - | 75705 | - | - | - | 75665 | - |
| -0.004  (-0.014, 0.006) | 0.438 | - | - | 0.026  (0.016, 0.037) | <0.001 | - | - | 0  (-0.01, 0.011) | 0.936 | - | - | 0.002  (-0.009, 0.012) | 0.716 | - | - |
| -0.166  (-0.177, -0.156) | <0.001 | - | - | -0.16  (-0.171, -0.148) | <0.001 | - | - | -0.208  (-0.219, -0.197) | <0.001 | - | - | -0.207  (-0.217, -0.196) | <0.001 | - | - |
| 0.015  (-0.01, 0.04) | 0.239 | 73077 | <0.001 | 0.022  (-0.003, 0.047) | 0.086 | 75378 | <0.001 | 0.022  (-0.003, 0.048) | 0.083 | 75690 | <0.001 | 0.023  (-0.002, 0.049) | 0.075 | 75649 | <0.001 |
| - | - | 73069 | 0.002 | - | - | 75367 | <0.001 | - | - | 75679 | <0.001 | - | - | 75637 | <0.001 |
| 0.045  (0.012, 0.079) | 0.009 | - | - | 0.049  (0.015, 0.083) | 0.005 | - | - | 0.048  (0.014, 0.082) | 0.005 | - | - | 0.049  (0.015, 0.083) | 0.005 | - | - |
| -0.01  (-0.043, 0.023) | 0.556 | - | - | -0.015  (-0.049, 0.018) | 0.377 | - | - | -0.015  (-0.049, 0.018) | 0.371 | - | - | -0.017  (-0.051, 0.017) | 0.319 | - | - |
| **N=18461, AIC=73058, p=0.017** | | | | **N=18464, AIC=75356, p=0.003** | | | | **N=18464, AIC=75666, p=0.003** | | | | **N=18464, AIC=75625, p=0.003** | | | |
| **Estimates** | | **ANOVA** | | **Estimates** | | **ANOVA** | | **Estimates** | | **ANOVA** | | **Estimates** | | **ANOVA** | |
| **B**  **(95%CI)** | **p-value** | **AIC** | **p-value** | **B**  **(95%CI)** | **p-value** | **AIC** | **p-value** | **B**  **(95%CI)** | **p-value** | **AIC** | **p-value** | **B**  **(95%CI)** | **p-value** | **AIC** | **p-value** |
| - | - | 73087 | - | - | - | 75393 | - | - | - | 75705 | - | - | - | 75665 | - |
| -0.004  (-0.014, 0.006) | 0.412 | - | - | 0.026  (0.016, 0.037) | <0.001 | - | - | 0  (-0.01, 0.01) | 0.979 | - | - | 0.002  (-0.009, 0.012) | 0.757 | - | - |
| -0.166  (-0.176, -0.155) | <0.001 | - | - | -0.159  (-0.171, -0.148) | <0.001 | - | - | -0.208  (-0.218, -0.197) | <0.001 | - | - | -0.206  (-0.217, -0.196) | <0.001 | - | - |
| 0.015  (-0.01, 0.04) | 0.248 | 73077 | <0.001 | 0.022  (-0.004, 0.047) | 0.092 | 75378 | <0.001 | 0.022  (-0.003, 0.047) | 0.089 | 75690 | <0.001 | 0.023  (-0.003, 0.048) | 0.08 | 75649 | <0.001 |
| - | - | 73069 | 0.002 | - | - | 75367 | <0.001 | - | - | 75679 | <0.001 | - | - | 75637 | <0.001 |
| 0.045  (0.012, 0.079) | 0.009 | - | - | 0.049  (0.015, 0.083) | 0.005 | - | - | 0.048  (0.014, 0.082) | 0.005 | - | - | 0.049  (0.015, 0.083) | 0.005 | - | - |
| -0.01  (-0.043, 0.024) | 0.565 | - | - | -0.015  (-0.048, 0.019) | 0.386 | - | - | -0.015  (-0.049, 0.019) | 0.383 | - | - | -0.017  (-0.051, 0.017) | 0.329 | - | - |
| **N=18363, AIC=72602, p<0.001** | | | | **N=18366, AIC=74860, p<0.001** | | | | **N=18366, AIC=75163, p<0.001** | | | | **N=18366, AIC=75128, p<0.001** | | | |
| **Estimates** | | **ANOVA** | | **Estimates** | | **ANOVA** | | **Estimates** | | **ANOVA** | | **Estimates** | | **ANOVA** | |
| **B**  **(95%CI)** | **p-value** | **AIC** | **p-value** | **B**  **(95%CI)** | **p-value** | **AIC** | **p-value** | **B**  **(95%CI)** | **p-value** | **AIC** | **p-value** | **B**  **(95%CI)** | **p-value** | **AIC** | **p-value** |
| - | - | 72667 | - | - | - | 74964 | - | - | - | 75271 | - | - | - | 75236 | - |
| -0.004  (-0.014, 0.006) | 0.44 | - | - | 0.026  (0.016, 0.037) | <0.001 | - | - | 0  (-0.01, 0.011) | 0.976 | - | - | 0.002  (-0.009, 0.012) | 0.754 | - | - |
| -0.166  (-0.177, -0.156) | <0.001 | - | - | -0.16  (-0.171, -0.148) | <0.001 | - | - | -0.208  (-0.219, -0.197) | <0.001 | - | - | -0.207  (-0.217, -0.196) | <0.001 | - | - |
| 0.011  (-0.015, 0.036) | 0.412 | 72658 | <0.001 | 0.016  (-0.009, 0.041) | 0.218 | 74949 | <0.001 | 0.016  (-0.01, 0.041) | 0.221 | 75256 | <0.001 | 0.017  (-0.009, 0.042) | 0.193 | 75221 | <0.001 |
| - | - | 72649 | 0.002 | - | - | 74938 | <0.001 | - | - | 75245 | <0.001 | - | - | 75208 | <0.001 |
| 0.046  (0.012, 0.08) | 0.007 | - | - | 0.05  (0.016, 0.084) | 0.004 | - | - | 0.05  (0.016, 0.084) | 0.004 | - | - | 0.05  (0.016, 0.085) | 0.004 | - | - |
| -0.009  (-0.043, 0.024) | 0.586 | - | - | -0.014  (-0.048, 0.02) | 0.42 | - | - | -0.014  (-0.048, 0.02) | 0.419 | - | - | -0.016  (-0.05, 0.018) | 0.349 | - | - |
